# Karyotyping and analysis of GNAS locus in intramuscular myxomas

**DOI:** 10.18632/oncotarget.14986

**Published:** 2017-02-01

**Authors:** Ioannis Panagopoulos, Ludmila Gorunova, Ingvild Lobmaier, Bodil Bjerkehagen, Sverre Heim

**Affiliations:** ^1^ Section for Cancer Cytogenetics, Institute for Cancer Genetics and Informatics, The Norwegian Radium Hospital, Oslo University Hospital, Oslo, Norway; ^2^ Centre for Cancer Biomedicine, Faculty of Medicine, University of Oslo, Oslo, Norway; ^3^ Department of Pathology, The Norwegian Radium Hospital, Oslo University Hospital, Oslo, Norway; ^4^ Faculty of Medicine, University of Oslo, Oslo, Norway

**Keywords:** intramuscular myxomas, cytogenetics, karyotyping, GNAS

## Abstract

Intramuscular myxoma is a benign soft tissue tumor about which very limited genetic information exists. We studied 68 intramuscular myxomas by means of chromosome banding analysis finding abnormal karyotypes in 21 of them. The most clearly nonrandom involvement was of chromosome 8 which was found gained in seven tumors (+8 was the sole change in five myxomas) and structurally rearranged in another two. Since mutation of the gene *GNAS* (20q13) has been implicated in the pathogenesis of both solitary and hereditary multiple myxomas, we assessed the transcription and mutation status of this gene in five tumors from which we had suitable RNA. All five intramuscular myxomas expressed biallelic transcripts. The mutated *GNAS* allele found in one tumor was also biallelically transcribed. In none of the five myxomas were maternally expressed transcripts detected. Collectively, the data suggest that intramuscular myxomas have acquired genetic abnormalities that often include chromosome 8 changes but may also involve alterations of *GNAS*. To what extent these aberrations are pathogenetically important, remains uncertain.

## INTRODUCTION

Intramuscular myxoma is a benign soft tissue tumor characterized by bland spindle-shaped cells embedded in hypovascular, abundantly myxoid stroma. The pathologic entity was first delineated by Enzinger in 1965 [1] and his description has since been amply confirmed by others [2–5]. Intramuscular myxoma is a rare tumor that occurs mostly in adults and shows a predilection for women (70%). The reported annual incidence is 0.10–0.13/100.000 [6, 7]. The vast majority of patients are asymptomatic with the tumor appearing as a painless, slowly enlarging, palpable, well-defined, round mass 2 to 15 cm in diameter [8]. Almost half of the tumors are found in the thigh. Intramuscular myxomas are usually solitary lesions not associated with any other clinical abnormalities [6]. Multiple tumors are rare but may be associated with fibrous dysplasia, also in the Mazabraud syndrome [9–12].

The tumors have a gelatinous, lobulated cut surface [6]. The fibrous capsule is usually incomplete with most lesions showing infiltration into adjacent musculature [6]. Histologically, intramuscular myxomas are hypocellular, hypovascular, intensely mucoid, and basophilic in hematoxylin-eosin stained preparations. The four main histologic components are interstitial mucin, sparse spindle-shaped cells, fine fibrillary reticulin fibers, and varying numbers of strands or trabeculae of fibrous tissue [6]. Other benign myxoid tumors that can be confused with intramuscular myxomas include myxolipoma, myxoid neurofibroma, nerve sheath myxoma, chondroma with myxoid change, and nodular fasciitis. More importantly, intramuscular myxoma can also be diagnostically confused with low grade myxoid sarcomas such as myxofibrosarcoma, low grade fibromyxoid sarcoma, myxoid liposarcoma, and extraskeletal myxoid chondrosarcoma [13].

Genetic information on intramuscular myxomas is very limited. Only one tumor studied by banding cytogenetics and having an abnormal karyotype has been reported; the tumor had trisomy 18 as the sole anomaly [14]. Interphase cytogenetic analysis by fluorescent *in situ* hybridization and DNA flow cytometry showed a normal DNA content and no indication of numerical chromosome aberrations in the four intramuscular myxomas studied by Aoki et al. [15].

Molecular genetic analyses of the *GNAS* gene (20q13) have revealed activating missense mutations, R201H and R201C, in exon 8 at codon 201 of the *GNAS* gene in both solitary intramuscular myxoma and the multiple intramuscular myxomas of Mazabraud syndrome [16–18]. The mutation frequency has varied from study to study depending on detection method. Okamato et al. [13] used single strand conformation polymorphism (SSCP) methodology to find point mutations in five of six intramuscular myxomas (three with and two without fibrous dysplasia), mutations which were subsequently confirmed by sequence analysis (three R201H and two R201C). Delaney et al. [16] detected mutations in 8 of 28 (29%) intramuscular myxomas by conventional PCR followed by mutation-specific restriction enzyme digestion whereas 17 of 28 (61%) mutations were detected using COLD-PCR followed by mutation-specific restriction enzyme digestion.

Walther et al. [18] used conventional PCR followed by direct sequencing to detect *GNAS* mutation in 23 out of 63 (36 %) intramuscular myxomas corresponding to 52 % R201C and 48 % R201H missense mutations.

Here we present our karyotypic analysis of intramuscular myxomas as well as analysis of the *GNAS* gene in five of the tumors.

## RESULTS

### Karyotyping and fluorescence *in situ* hybridization (FISH) analyses

Abnormal karyotypes were found in 21 out of 68 tumors, 12 from female and 9 from male patients (Tables 1 and 2). Numerical aberrations only were seen in 12 tumors, whereas both numerical and structural rearrangements were found in 9. Almost all abnormal clones were pseudodiploid or near-diploid whereas one clone in case 11 was hyperhaploid. The vast majority (90 %) of cytogenetically abnormal tumors had simple karyotypes (1–3 chromosome changes) with only two tumors having complex karyotypes (6–7 aberrations) (cases 11 and 14). Two tumors (cases 3 and 20) had two cytogenetically unrelated clones; one with structural, the other with numerical chromosome aberrations.

**Table 1:** Information on the cytogenetically analyzed myxomas

Samples	Analyzed Cases	Abnormalkaryotypes	Only numericalaberrations	Both numerical andstructural aberrations
Female	44	12	6	6
Male	24	9	6	3
Total	68	21	12	9

**Table 2:** Clinicopathological data on the intramuscular myxomas with abnormal karyotypes

Cases	Sex/Age	Site	Largest diameter (cm)	Karyotype
1	F/36	Left shoulder	2.5	47,XX,+3[2]/46,XX[23]
2	M/54	Right thigh	3	47,XY,+8[2]/46,XY[22]
3	F/75	Left shoulder	2	47,XX,+7[2]/46,XX,del(6)(q21)[2]/46,XX[11]
4	F/61	Right thigh	3	47,XX,+8[3]/46,XX[22]
5	F/69	Right shoulder	7.5	47,XX,+8[3]/46,XX[21]
6	F/47	Left thigh	Not available	46,XX,del(8)(q13 or q13q22)[6]/46,XX[9]
7	M/68	Right Shoulder	6	45,X,-Y[3]/46,XY[23]
8	F/55	Back	5.5	47,XX,+8[2]/46,XX[14]
9	F/68	Right thigh	3	47,XX,+8[2]/46,XX[22]
10	F/53	Right shoulder	1.8	46,XX,der(1)inv(1)(p32q42)t(1;4)(q42;q21),der(4)t(1;4) [3]/46,XX[22]
11	M/54	Left thigh	3.5	29,X,-Y,+5,+7,+8,+12,+18,+19[20]/47,X,-Y,+4,+10[12]/46,XY[4]
12	F/48	Right shoulder	4	47,XX,+del(22)(q11)[16]
13	M/34	Left chest wall	4.7	46,XY,t(7;15)(q32;q15∼22),t(11;17)(q23;q23)[15]
14	F/67	Right upper arm	3	45∼46,XX,add(1)(p22),-5,der(9)t(5;9)(q11;p21),-17,+1∼2mar[cp7]/46,XX[3]
15	M/73	Left shoulder	2	46,XY,t(2;8)(p21;q13∼21),t(10;11)(p14∼15;q12∼13)[5]/46,XY[5]
16	M/56	Right shoulder	3	47∼48,XY,+7[2][cp2]/46,XY[23]
17	F/54	Left thigh	4.2	47,XX,+X[2]/46,XX[23]
18	M/46	Right thigh	3.5	47,XY,+7[3]/46,XY[12]
19	M/49	Abdominal wall	2.6	45∼47,XY,del(6)(q21q23),+8,tas(14;17)(pter;qter)[cp11]/46,XY[2]
20	F/74	Left thigh	3.6	46,XX,add(19)(p13),-21,+r[7]/47,XX,+7[2]/46,XX[9]
21	M/70	Right thigh	6.2	48,XY,+7,+9[5]/46,XY[20]

**Figure 1:**
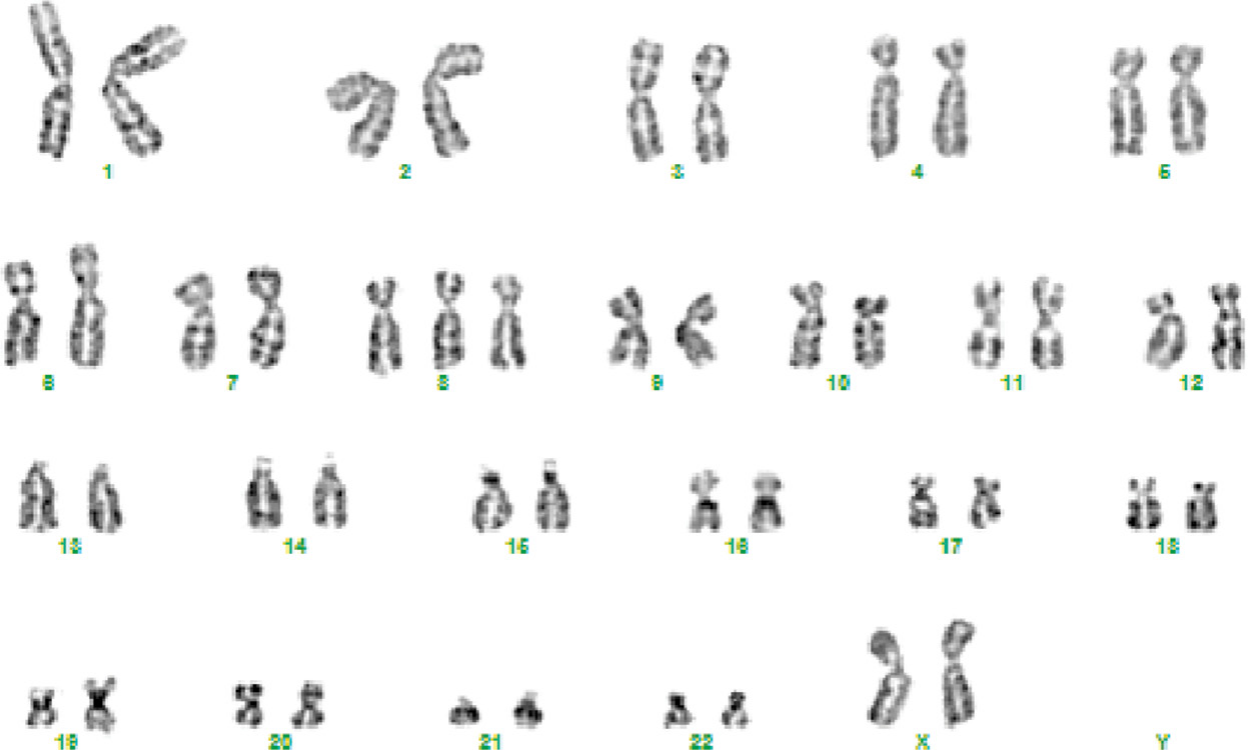
Karyotype of case 8 showing trisomy 8 as the sole anomaly

The most clearly nonrandom involvement was of chromosome 8 seen in 9 tumors, 7 of which showed trisomy 8 whereas 2 had structural aberrations. Trisomy 8 was the sole anomaly in 5 myxomas (Figure 1). In both myxomas with structural aberrations of chromosome 8, bands 8q13-q22 were involved: one tumor (case 6) had a del(8)(q13 or q13q22) as the only cytogenetic change whereas the other (case 15) had the translocation t(2;8)(p21;q13∼21) as the sole change (Table 2). Chromosome 7 was the second most frequently involved showing aberration in 6 tumors: 5 with numerical changes (trisomy 7; cases 3, 11, 16, 18, and 21) and one with a translocation t(7;15)(q32;q15∼22) (case 13). A del(6)(q21) was found in two tumors (cases 3 and 19).

In case 12, the karyotype was initially 47,XX,+mar. FISH with painting probes for chromosomes 19 and 22 showed that the marker contained material from chromosome 22 (data not shown). FISH with a breakapart probe for *EWSR1* showed that the *EWSR1* locus was not on the marker chromosome, nor was there any splitting of it (data not shown).

### Analysis of *GNAS* expression and mutation

**Figure 2:**
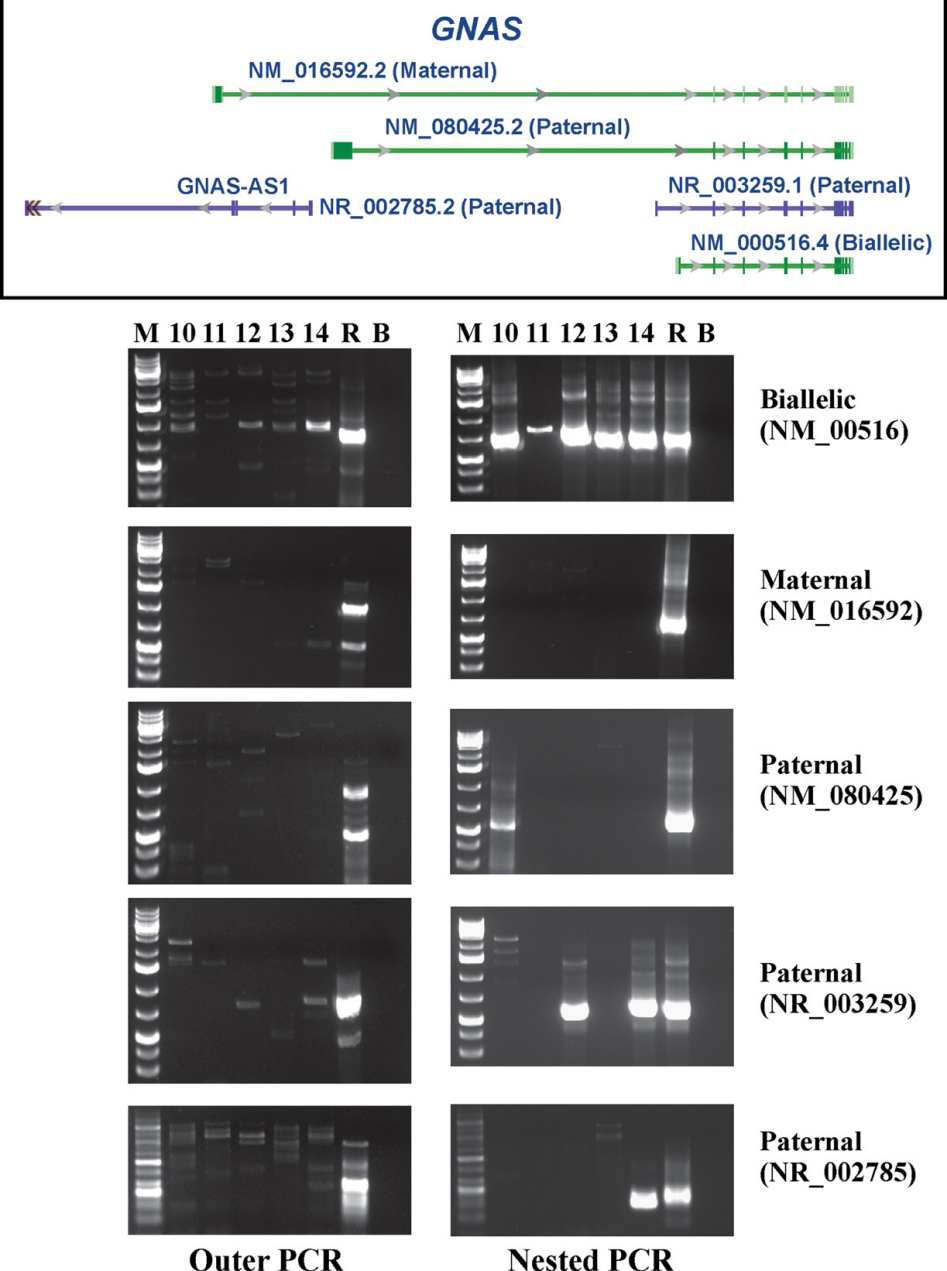
RT-PCR analysis for the expression of the biallelically, maternally, and three paternally expressed GNAS transcripts in cases 10–14 R is human universal reference total RNA. B is blank. M is 1kb Plus DNA ladder (GeneRuler, Fermentas).

Expression analysis of the *GNAS* gene was for reasons of stored material shortage possible for cases 10–14 only (Figure 2). The *GNAS* locus has a highly complex imprinted expression pattern giving rise to transcripts (including non-coding ones) that are maternally, paternally, or biallelically expressed [19–21].

Outer and nested RT-PCR amplified the biallelically expressed transcript (NM_000516) in the examined tumors (Figure 2). In none of the tumors was the maternally expressed transcript amplified (NM_016592). The paternally expressed transcript with accession number NM_080425 was detected in case 10 (Figure 2), whereas the paternally expressed transcript with accession number NR_003259 was found in cases 12 and 14. Only tumor 14 expressed the paternally expressed transcript with accession number NR_002785 (Figure 2).

**Figure 3:**
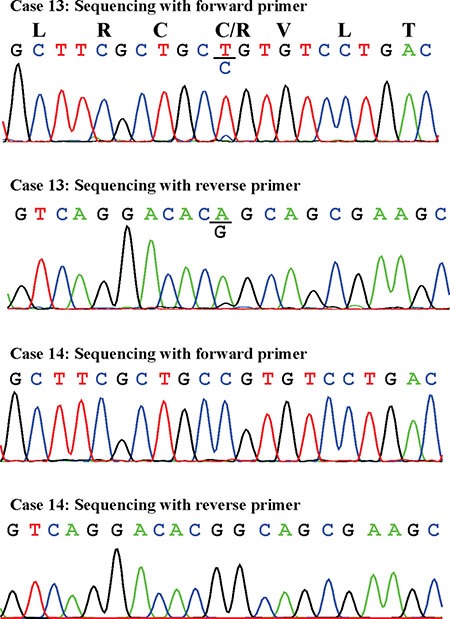
Partial sequence chromatogram of the cDNA fragment showing the mutation R201C in case 13 and the normal R201 in case 14 Sequences with both the forward and reverse primers are shown.

The PCR products amplified in nested PCR using the primer set GNAS-379F1+GNAS-1040R1 corresponded to the biallelically expressed transcript with accession number NM_000516. Direct sequencing of these PCR products detected the R201C mutation in case 13 only (Figure 3). No mutations were found at codon 227.

## DISCUSSION

Information about the acquired genomic abnormalities of tumor cells, be it at the chromosomal or molecular level of resolution, is a powerful adjunct to microscopic tumor features in diagnostic pathology. The same information is also crucial to obtaining any deep understanding of tumorigenesis. The present study describes the largest series of cytogenetically analyzed intramuscular myxomas to date. It proves that acquisition of clonal chromosome aberrations is an integral part of the disease process inasmuch as 21 out of 68 intramuscular myxomas were shown to have clonal cytogenetic aberrations. Abnormalities of chromosome 8, mainly trisomy, followed by trisomy for chromosome 7 were the most common aberrations (Tables 1 and 2). Trisomy 8 is common also in cancer and may be found both alone and together with other aberrations [22]. Trisomy 8 as the sole abnormality occurs particularly in acute myeloid leukemia (AML) and myelodysplastic syndromes (MDS), in 5–10% of cytogenetically abnormal cases [23]. The etiology, molecular mechanisms, and pathogenetic consequences behind this and other numerical chromosome aberrations remain unknown. However, a recent study indicated that AMLs with trisomy 8 as sole anomaly have distinct gene and microRNA-expression signatures [24]. In solid tumors, polysomy 8 was found in different diagnostic entities such as desmoid-type and superficial fibromatosis, clear cell sarcomas with t(12;22)(q13;q12), Ewing sarcomas, myxoid liposarcomas, synovial sarcomas, hepatoblastomas, Wilms’ tumor, and colorectal cancer [22]. Again, the etiology and pathogenetic consequences behind the change are unknown. The same goes for trisomy 7 which is also frequently seen in various both neoplastic and non-neoplastic disease lesions presenting as solid tumors [22, 25].

Intramuscular myxoma, and in particular its cellular variant, shows considerable overlapping histological features with grade I myxofibrosarcoma [26]. The “Mitelman Database of Chromosome Aberrations and Gene Fusions in Cancer” (https://cgap.nci.nih.gov/Chromosomes/Mitelman; database last updated on August 11, 2016) contains cytogenetic information on 84 myxofibrosarcomas of various grades. None of them had trisomy 8 or trisomy 7 as the sole cytogenetic abnormality.

Willems et al. [27] studied the genetic alterations and composition of extracellular matrix of intramuscular myxoma and grade I myxofibrosarcoma. Of the ten examined intramuscular myxomas, four did not have cytogenetic data whereas six had a normal karyotype. Of the ten myxofibrosarcomas, four did not have cytogenetic data, one had a normal karyotype, another had a balanced t(9;12) translocation, a third had monosomy of chromosome 21, and three had complex karyotypes. Moreover, five myxomas had mutation at codon 201 of *GNAS*. Neither myxomas nor myxofibrosarcomas had mutations of *KRAS* codon 12/13 or *TP53*, nor was there any significant difference in *FOS* expression between intramuscular myxoma and grade I myxofibrosarcoma. The only difference found between intramuscular myxoma and grade I myxofibrosarcoma was in the expression of decorin, a matrix proteoglycan, which was expressed in myxofibrosarcomas but not in myxomas [27]. Both decorin immunoreactivity and mRNA expression of *DCN* (the gene coding for decorin) were positive in the former but not in the latter [27]. In a recent study, on the other hand, Cates et al. [28] saw no difference in decorin immunoreactivity between myxoma and myxofibrosarcoma. In fact, none of the 19 potential diagnostic markers tested distinguished myxoma from myxofibrosarcoma [28]. The authors concluded that the distinction between these tumors must still be made based on morphologic criteria.

The *GNAS* locus has a highly complex imprinted expression pattern giving rise to maternally, paternally, and biallelically expressed transcripts (including non-coding ones) that are derived from four alternative promoters and 5′ exons [19–21]. An antisense transcript is produced from an overlapping locus on the opposite strand. One of the transcripts produced from this locus as well as its antisense transcript are paternally expressed noncoding RNAs that may regulate imprinting in the region (http://www.ncbi.nlm.nih.gov/gene/2778). Alternative splicing of downstream exons is also observed, resulting in different forms of the stimulatory G-protein alpha subunit, a key element in the classical signal transduction pathway linking receptor-ligand interactions with the activation of adenylyl cyclase and a variety of cellular responses (http://www.ncbi.nlm.nih.gov/gene/2778). In the present study, we found expression of the biallelic transcript with accession number NM_000516 which codes for guanine nucleotide binding protein alpha s long (GNASL), also known as alpha-S2, a form of the G-protein alpha subunit (http://www.ncbi.nlm.nih.gov/gene/2778). In contrast, none of the tumors expressed the maternally expressed transcript (accession number NM_016592). This transcript encodes secretogranin VI (SCG6, also known as NESP55) which localizes to large secretory vesicles of endocrine cells and neurons. Its coding region is within the most 5′ exon and does not overlap the coding regions used by other transcripts; thus, SCG6 has no similarity to isoforms of the G-protein alpha subunit. No consistent expression pattern was found for the three paternally expressed transcripts NM_080425, NR_003259, and NR_002785 (Figure 1).

Mutation analysis of the biallelically expressed transcript identified the mutation R201C in one out of five samples. This is in agreement with findings presented in previous reports that only some myxomas have mutations of codon R201 [16–18]. None of the examined myxomas had mutation of codon Q227 which was found in a small proportion of fibrous dysplasia tumors [29].

Apart from myxomas, R201C, R201H, and Q227R mutations of GNAS protein were found also in a variety of other neoplasms such as tumors of the kidney, thyroid, pituitary, leydig cells, adrenal cortex, and large bowel [30]. These mutations are thought to inhibit guanosine triphosphate hydrolysis resulting in constitutive activation of the stimulatory G-protein alpha subunit and its effector adenylate cyclase, leading to autonomous synthesis of cAMP [31, 32]. To study the role of *GNAS* in intestinal tumorigenesis, Wilson et al. [30] placed GNAS R201C under the control of the A33-antigen promoter (Gpa33) which is almost exclusively expressed in the intestines. GNAS R201C was found to cause augmentation of both the Wnt and ERK1/2 MAPK pathways in the intestinal epithelium of mice and the mutation co-operated with inactivation of Apc in intestinal tumor formation *in vivo* [30]. Collectively, the data suggest that mutations of GNAS can modify cell growth and may be oncogenic; however, how *GNAS* functions as an oncogene in the various tumors remains unclear.

In summary, our study showed that intramuscular myxomas are characterized by acquired numerical but occasionally also structural chromosome rearrangements, express the biallelic *GNAS* transcript but not the maternally expressed transcript, and that the mutated allele of *GNAS* is biallelically transcribed in the tumor cells.

## MATERIALS AND METHODS

### Patients

The material consisted of 68 samples from tumors diagnosed as intramuscular myxomas (Table 1), all surgically removed at The Norwegian Radium Hospital between 2000 and 2016. The patients, 46 females and 26 males, were from 34 to 78 years old with a median age of 55. The study was approved by the regional ethics committee (Regional komité for medisinsk forskningsetikk Sør-Øst, Norge, http://helseforskning.etikkom.no). Written informed consent was obtained from the patients to publication of the case details. The ethics committee's approval included a review of the consent procedure. All patient information has been de-identified.

### G-banding, karyotyping, and FISH

Fresh tissue from a representative area of the tumor was received and analyzed cytogenetically as part of our diagnostic routine. The samples were disaggregated mechanically and enzymatically with collagenase II (Worthington, Freehold, NJ, USA). The resulting cells were cultured and harvested using standard techniques. Chromosome preparations were G-banded with Wright's stain (SigmaAldrich; St Louis, MO, USA) and examined. Metaphases were analyzed and karyograms prepared using the CytoVision computer assisted karyotyping system (Leica Biosystems, Newcastle, UK). The karyotypes were described according to the International System for Human Cytogenetics Nomenclature [33].

For case 12 (see below, Table 2), FISH was performed on metaphase spreads using painting probes for chromosomes 19 and 22 as well as an *EWSR1* breakapart probe (Cytocell, Cambridge, UK).

### RNA extraction and cDNA synthesis

Frozen (–80°C) tumor tissue adjacent to that used for cytogenetic analysis and histologic examination was available for cases 10-14 (Table 2). Total RNA was extracted using Trizol reagent according to the manufacturer's instructions (Life Technologies, Oslo, Norway). cDNA was synthesized from this in a 20 μL reaction volume using iScript Advanced cDNA Synthesis Kit for RT-qPCR according to the manufacturer's instructions (Bio-Rad Laboratories).

### Analysis for *GNAS* expression

Nested RT-PCR was performed to assess the expression of *GNAS*. The primers used for PCR amplification and sequencing are listed in Table 3. The primer combinations, target fusion transcripts, and results of PCR amplifications are shown in Table 4. Human Universal Reference Total RNA was used as control (Clontech Laboratories, TaKaRa-Bio Group, Europe/SAS, Saint-Germain-en-Laye, France). According to the company's information, it is a mixture of total RNAs from a collection of adult human tissues chosen to represent a broad range of expressed genes. Both male and female donors are represented. Four μL of cDNA (1 μL for the control sample) were used as template and amplified using the outer primer combination. One μL of the amplified products was used as template in nested PCR. For both outer and nested PCRs, the 25 μL reaction volume contained 12.5 μL of Premix Taq (TaKaRa-Bio Europe/SAS, Saint-Germain-en-Laye, France), template, and 0.4 μM of each of the forward and reverse primers (Tables 3 and 4). The PCRs were run on a C-1000 Thermal cycler (Bio-Rad Laboratories). PCR cycling consisted of an initial step of denaturation at 94°C for 30 sec followed by 35 cycles of 7 sec at 98°C, 30 sec at 60°C, 2 min at 72°C, and a final extension for 5 min at 72°C. Three μL of the PCR products were stained with GelRed (Biotium), analyzed by electrophoresis through 1.0% agarose gel, and photographed. DNA gel electrophoresis was performed using lithium borate buffer [34].

**Table 3:** Primers used for PCR amplification and sequencing

Oligo Name	Sequence (5′->3′)	Position	Accession number
GNAS-356F1	CATGGGCTGCCTCGGGAACAG	356–376	NM_000516.4
GNAS-379F1	AGACCGAGGACCAGCGCAACG	379–399	NM_000516.4
GNAS-459F1	CAGGTCTACCGGGCCACGCAC	459–479	NM_000516.4
GNAS-1088R1	GCTGCTGGCCACCACGAAGATG	1109–1088	NM_000516.4
GNAS-1040R1	GATCCACTTGCGGCGTTCATCG	1061–1040	NM_000516.4
GNAS-NR-193F1	AGGCGCTGCCTTGCGTGTGA	193–212	NR_003259
GNAS-NR-213F1	GTGCACCTCACTCACATGTGCTGGA	213–237	NR_003259
GNAS-1016F1-Mat	CGTCGCTGCAAGCCAAAGAAGC	1016–1037	NM_016592.2
GNAS-1089F1-Mat	CCATCCGGCGTCACTAATGGAGG	1089–1111	NM_016592.2
GNAS-2315F1-Pat	AGATGGGCTACATGTGTACGCACCG	2315–2339	NM_080425.2
GNAS-2223F1-Pat	ACAGATGCGCAAAGAAGCCCTGG	2223–2245	NM_080425.2
GNAS-841F1	GCTACGAACGCTCCAACGAGTA	841–862	NM_000516.4
GNAS-921F1	GACTATGTGCCGAGCGATCA	921–940	NM_000516.4
GNAS-982R1	TGTCCACCTGGAACTTGGTCT	1002–982	NM_000516.4
GNAS-AS1-NR-249F	GCAAGAAGATTTCCAGGGCTGGGA	249–272	NR_002785.2
GNAS-AS1-NR-312F	GGAGCAGCCCAGGATGGATAAGGA	312–335	NR_002785.2
GNAS-AS1-NR-574R	AACGGCAGCAATCTGGTAACGCAC	597–574	NR_002785.2
GNAS-AS1-NR-652R	CGGCCATTTTCAGCACGGGTAGA	674–652	NR_002785.2

**Table 4:** Primer combinations for outer and nested PCR amplification of GNAS transcripts, accession number of the sequence and expression of the target amplification transcript

Outer PCR	Nested PCR	Amplification sequence	Expression
GNAS-356F1 +GNAS-1088R1	GNAS-379F1 +GNAS-1040R1	NM_000516.4	Biallelic
GNAS-1016F1-Mat +GNAS-1088R1	GNAS-1089F1-Mat +GNAS-1040R1	NM_016592.2	Maternal
GNAS-2223F1-Pat +GNAS-1088R1	GNAS-2315F1-Pat +GNAS-1040R1	NM_080425.2	Paternal
GNAS-NR-193F1 +GNAS-1088R1	GNAS-NR-213F1 +GNAS-1040R1	NR_003259.1	Paternal
GNAS-AS1-NR-249F +GNAS-AS1-NR-652R	GNAS-AS1-NR-312F +GNAS-AS1-NR-574R	NR_002785.2	Paternal

### Analysis for *GNAS* mutation

The PCR products which were amplified in nested PCR using the primer set GNAS-379F1+GNAS-1040R1 were purified using the Thermo Scientific GeneJET PCR purification Kit (Fisher Scientific, Oslo, Norway) and direct sequencing was performed using the light run sequencing service of GATC Biotech (https://www.gatc-biotech.com/en/index.html). The BLAST (http://blast.ncbi.nlm.nih.gov/Blast.cgi) program was used for computer analysis of sequence data against the reference sequence with accession number NM_000516.4.
